# Thermal Imaging as a New Perspective in the Study of Physiological Changes in Pregnant Women—A Preliminary Study

**DOI:** 10.3390/jcm14175998

**Published:** 2025-08-25

**Authors:** Karolina Rykała, Agnieszka Szurko, Daria Wziątek-Kuczmik, Agnieszka Kiełboń, Manuel Sillero-Quintana, Armand Cholewka, Teresa Kasprzyk-Kucewicz

**Affiliations:** 1 Faculty of Science and Technology, University of Silesia, 40-055 Katowice, Poland; karolinarykala9@gmail.com (K.R.); agnieszka.szurko@us.edu.pl (A.S.); armand.cholewka@us.edu.pl (A.C.); teresa.kasprzyk-kucewicz@us.edu.pl (T.K.-K.); 2 Department of Cranio-Maxillofacial Surgery, Faculty of Medical Sciences, Medical University of Silesia, 10-055 Katowice, Poland; 3 Faculty of Physical Activity and Sports Sciences, Universidad Politécnica de Madrid, 28040 Madrid, Spain; manuel.sillero@upm.es

**Keywords:** thermal infrared imaging, pregnancy, trimester of pregnancy, body temperature of a pregnant woman

## Abstract

**Background/Objectives**: This study aimed to examine the dynamic thermal variations that occur in the posterior body regions of pregnant women by employing thermal imaging techniques. **Methods**: The study involved the participation of 34 women in various stages of pregnancy. The skin temperature (Tsk) distribution in specific body areas, including the spinal region and lower limbs, was analyzed under standard conditions. **Results**: The most considerable increase in body temperature (Tsk) recorded in female volunteers was achieved during the second trimester of pregnancy in physiologically stressed areas, such as the upper back (0.4 °C), lower back (0.77 °C), thighs (0.94 °C) and calves (0.32 °C). Contrastingly, a decrease in Tsk of noteworthy magnitude was observed in all body regions during the third trimester, with an average decrease of 1.7 °C. The lower back’s most substantial decrease was observed (1.95 °C). Furthermore, a disparity was observed in the Tsk distribution of the calves, with the highest ∆T_mean_ value recorded at approximately 0.5 °C, and the thighs exhibiting a ∆T_mean_ value of 0.25 °C. **Conclusions**: Preliminary studies have demonstrated the potential of thermal imaging as a reliable and safe method to support prenatal diagnosis. Its application can facilitate the early detection of health complications, including inflammatory states or posture and circulatory system disorders, thereby enhancing the standard of prenatal care.

## 1. Introduction

During pregnancy, a woman’s body undergoes numerous anatomical and physiological changes due to fetal development, often causing physical discomfort. The gradual transformations encompass the musculoskeletal system and the functions of the respiratory, digestive, and endocrine systems. In addition, cardiovascular changes increase circulating blood volume, cardiac output, and heart rate. Water retention is also observed, contributing to increased body weight and circulation problems. Metabolic changes, including a substantial increase in insulin secretion, contribute to increasing the fat storage capacity [1,2]. Thermoregulatory processes are also modified during the development of pregnancy, which is related to the action of pregnancy hormones and the increase in dermal blood flow [3,4].

Several anatomical and physiological changes during pregnancy make pregnant women more susceptible to strains and other specific health problems. One of the most frequent complaints during pregnancy is low back pain (LBP), which is a prevalent complaint during pregnancy. Statistically, it affects between 30% and almost 80% of women. LBP is often associated with lower limb pain [5,6]. The other problems are visible in the asymmetry in the limbs’ blood flow and water retention.

The most common tools to monitor and care for pregnant women are biochemical blood and urine tests, ultrasound, and cardiotocography (CTG) performed during the third trimester or after delivery [7,8].

In recent years, there has been a rapidly growing interest in using thermal imaging in medicine [9]. Thermal imaging provides valuable data, such as thermal maps of different body parts, from which characteristic temperature parameters can be calculated. The potential for using thermal imaging as an additional technique to other diagnostic methods has been recognized in several conditions, including the diagnosis of inflammatory processes, cancer, breast disease, peripheral vascular disease, dermatological conditions, Raynaud’s disease, trophic ulcers of the lower extremities, and many other skin and soft tissue lesions [9,10,11,12,13,14,15,16,17]. It is worth noting that thermal imaging has already been used to study pregnant animals [17,18].

The few publications on pregnant women mainly provide information on skin temperature (Tsk) changes in specific parts of the body or an overall assessment of the position of the placenta [19,20,21,22]. Nevertheless, these reports support using infrared thermography to monitor the Tsk of specific areas of the pregnant woman’s body [22,23,24]. However, there are few studies on thermal imaging that focus on a comprehensive evaluation of the whole body of a pregnant woman [22,24]. Moreover, there are no quantitative temperature analyses during the whole pregnancy period. However, it is known that fetal temperature is strongly dependent on fetal metabolism, maternal temperature, and uterine blood flow. The fetal temperature is usually about 0.5 °C higher than the maternal internal temperature due to heat production during increased metabolism [1,20,25]. The umbilical circulation transfers 84.5% of the fetus’s heat to the mother. The remaining heat generated by the fetus is lost through contact with the amniotic membrane and then with the uterine wall and the mother’s abdomen [20,26]. Thermal changes in the maternal and fetal bodies are the basis for using infrared thermal imaging as a safe, non-invasive, and low-cost complementary method for standard diagnostic imaging. In addition, it is noteworthy that assessing the Tsk distribution of the expectant mother’s body in the early stages of pregnancy could provide important health information.

The research conducted is a pilot measurement. During the study, changes in Tsk distribution and thermal parameter values in women at all stages of pregnancy were monitored using a thermal imaging camera. The areas of the body that undergo the most significant functional changes during pregnancy were analyzed. The long-term aim of the measurements is to monitor pregnant women for the early detection of pathological changes that most commonly affect women during pregnancy. In essence, thermal imaging studies could help diagnose and even predict the occurrence of stress pains, which are directly related to changes in the symmetry of Tsk distribution within individual body regions observed on thermal images. The non-invasiveness, relatively low cost and ease of performing such an examination in an outpatient clinic or at the patient’s home are undoubted advantages of thermal imaging as a new screening tool.

## 2. Materials and Methods

The study group consisted of 14 volunteers at different stages of pregnancy. The data collected were divided into trimesters according to the physiological stage of pregnancy development: 9 volunteers were examined in the 1st trimester (1 to 13 weeks of pregnancy), 14 volunteers were examined in the 2nd trimester (14 to 27 weeks of pregnancy), and 11 volunteers were examined in the 3rd trimester (28 to 40 weeks of pregnancy). It must be considered that three volunteers were examined in each of the three trimesters, while the others were mainly discussed in two or one trimester.

Preparation for the thermographic examination included 30 min of acclimatization to the conditions of the examination room (temperature 22 ± 1 °C, humidity 50–60%) by sitting quietly with the parts of the body to be imaged exposed. In addition, volunteers were asked to refrain from physical activity for at least 10 h before the examination, to refrain from using analgesics or antipyretics for the previous 24 h, and to refrain from using creams, oils, and ointments for at least 12 h before the examination. Hot drinks or stimulants were also not allowed.

Thermal imaging was performed using a Flir Systems T540 thermal imaging camera with a resolution of 464 × 348 pixels and a sensitivity of 30 mK. The camera was set on a tripod at a distance of 1.5 m ± 0.1 m from the patient. The research protocol was in accordance with the guidelines of the Declaration of the World Medical Association in Helsinki.

The study utilized thermal imaging technology to acquire a series of thermal images, which were subsequently subjected to rigorous analysis. A pivotal step in this analysis entailed the isolation of specific regions of interest (ROIs) and the determination of the minimum, maximum and average temperature values for these regions. This methodological approach facilitates a more precise interpretation of the data obtained. It can contribute to a more comprehensive understanding of the physiological processes occurring in the female body during pregnancy.

**Figure 1:**
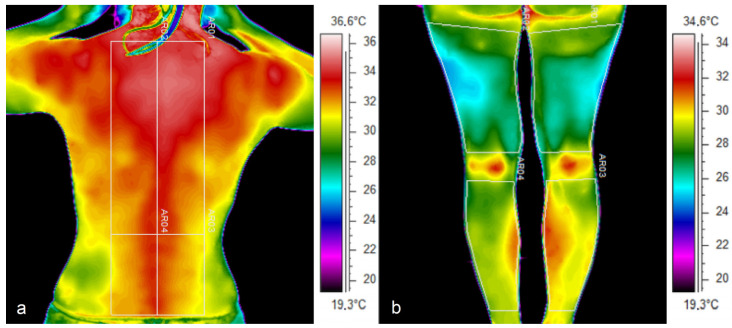
Thermal images of the pregnant woman in the 1st trimester, where (**a**) dorsal part, (**b**) posterior surface of the lower limbs with marked regions of interest.

The average T_sk_ assessment was made for symmetrical areas of the lower limbs, such as the calves, thighs and upper back from Th5 to Th12 and the lower back from L5 to S1. The lines of symmetry were defined by the spine and the body’s midline (linea mediana posterior). Figure 1 shows thermograms of the different parts of the body studied for a representative pregnant woman at 5 weeks of gestation and indicates the regions of interest (ROIs) from which Tsk parameters have been derived for subsequent analyses.

The statistical analysis was conducted using IBM SPSS Statistics software, version 14.1.0. The analysis was performed using parametric ANOVA/MANOVA tests. To assess the effect of time and body location on the variability of the average chosen body (ROIs) surface temperature, an analysis of variance (ANOVA) with repeated measurements was used. The data obtained for the left and right sides of the woman’s body were analyzed separately. Post hoc Bonferroni tests were also performed due to the limited number of measurements, assessing the effect of pregnancy progression (time factor) and the location of the measured temperature (temperature comparisons between individual ROIs). Before the analysis, the assumptions of normality, homogeneity of distribution, and sphericity were verified using Mauchly’s test. As part of the analysis of variance (ANOVA) tests, trend analysis was used with linear and quadratic models to identify the nature of temperature changes over time. Furthermore, a multivariate analysis of variance (MANOVA) was conducted to evaluate the combined impact of the ‘time’ factor on a set of dependent variables (temperature in multiple ROIs simultaneously). The level of statistical significance was set at *p* < 0.05.

## 3. Results

**Figure 2:**
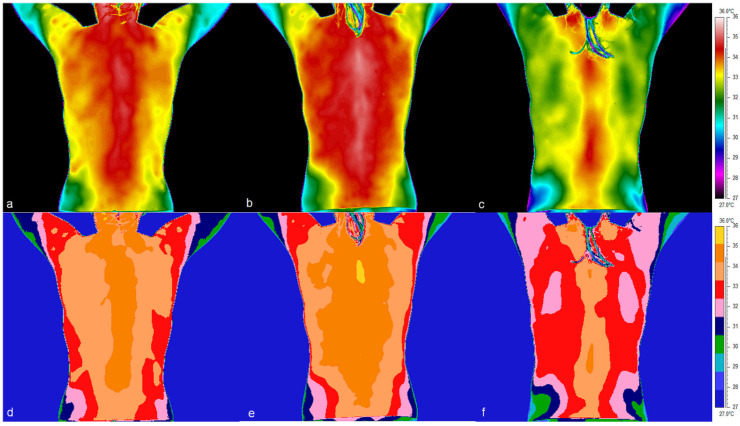
Comparison of thermal images in two color scales of the dorsal part taken in a female volunteer: (**a**,**d**) at 9 weeks of pregnancy—1st trimester, (**b**,**e**) at 22 weeks of pregnancy—2nd trimester, (**c**,**f**) at 40 weeks of pregnancy—3rd trimester. Images (**a**–**c**) are presented using the rainbow scale, while (**d**–**f**) are presented using the medical scale.

Thermal images of the back of a representative female volunteer examined in the first, second and third trimesters of pregnancy clearly show a developing asymmetry in the distribution of Tsk in the paravertebral region (Figure 2). This asymmetry intensified in the second (Figure 2b,e) and third trimester (Figure 2c,f) when the volunteer reported a diagnosis of sciatica with pain in the lumbosacral region of the spine radiating to the left leg. The medical scale enables clear visualization of the outlined thermal asymmetries.

**Figure 3:**
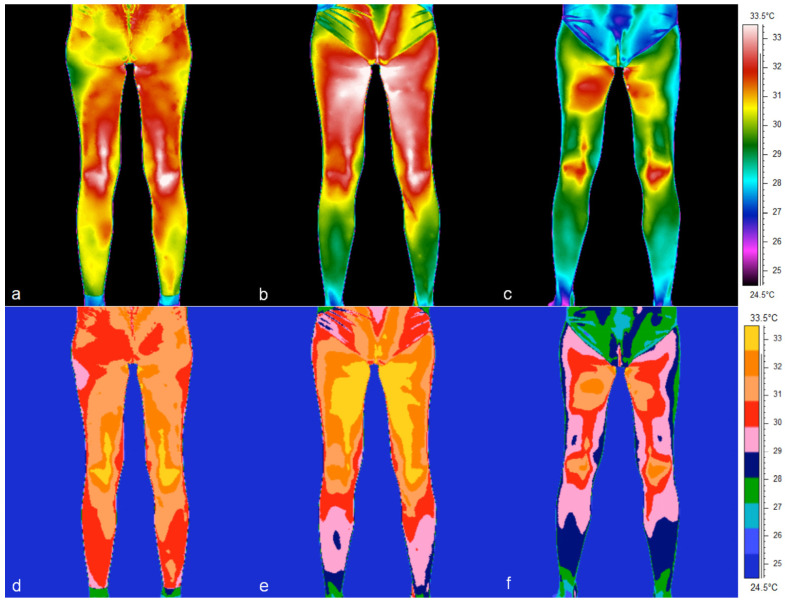
Comparison of thermograms in two color scales of the posterior surface of the lower limbs taken in a female volunteer: (**a**,**d**) at 9 weeks of pregnancy—1st trimester, (**b**,**e**) at 22 weeks of pregnancy—2nd trimester, (**c**,**f**) at 40 weeks of pregnancy—3rd trimester. Images (**a**–**c**) are presented on the rainbow scale, while (**d**–**f**) are presented on the medical scale.

**Figure 4:**
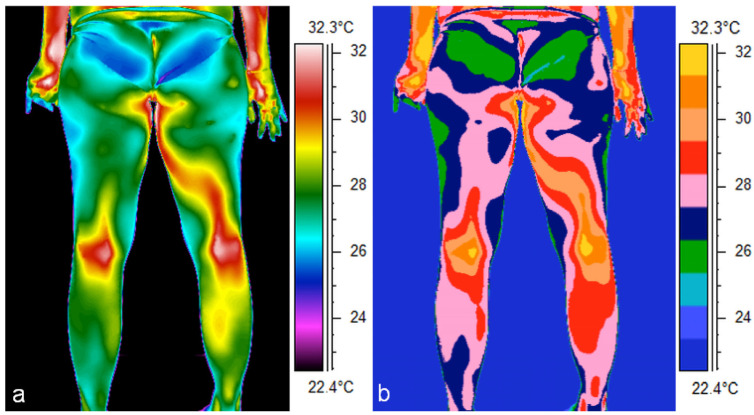
Comparison of thermograms in two color scales of the posterior surface of the lower limbs taken in the 3rd trimester of a female volunteer, where (**a**) rainbow scale, (**b**) medical scale.

**Figure 5:**
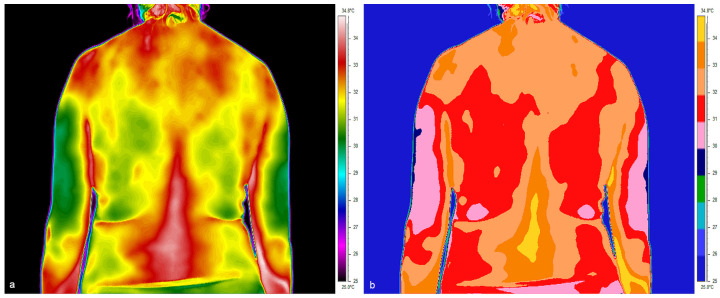
Thermal images presented in two color scales of the dorsal part taken in the 3rd trimester of a female volunteer, where (**a**) rainbow scale, (**b**) medical scale.

In addition, there is an apparent asymmetry in the distribution of Tsk in the posterior lower limb of a particular volunteer, manifesting itself in both the thigh and calf regions (Figure 3). In the images, both on the rainbow and medical scales, an increase in thigh temperature in the second trimester and a decrease in the third trimester can easily be seen (Figure 2 and Figure 3). Analysis of the thermal images taken for a representative volunteer indicated a systematic decrease in the mean Tsk values in the calf and in the dorsalarea between the 1st and 3rd trimesters (Figure 4 and Figure 5).

The mean temperatures from the symmetrical and corresponding body areas on the right and left sides were calculated based on the obtained thermal images. The attention was mainly focused on those body parts that physiologically have to adapt significantly to the new conditions resulting from the developing pregnancy. Thus, Table 1 presents the mean values of Tsk measured for the upper part of the spine (corresponding to the thoracic spine, i.e., Th5 to Th12) of pregnant women in the first, second and third trimesters of pregnancy (Table 1). In trimester 1, the mean temperature values were 33.67 °C on the left and 33.72 °C on the right. The temperature difference occurring between the body sides was also calculated. In trimester I of pregnancy, the maximum mean temperature difference between the sides of the body was observed in the paracervical region at 0.17 °C (∆T_L/R_). Conversely, in the subsequent trimester II, a significant increase in the mean temperature was observed in the supraspinal regions, with proximal temperatures reaching 34.09 °C on the left side and 34.1 °C on the right side. In contrast, during the second trimester, a smaller disparity in temperature distribution between the sides of the body was observed in the proximal spinal region (∆T_L/R_ = 0.11 °C). In the third trimester, the lowest mean temperature values of 32.29 °C and 32.18 °C were recorded for the left and right sides, respectively, while the ∆T_L/R_ was 0.18 °C.

Comparable calculations were made for the lower back (L5 to S1), and the values are summarized in Table 2. This region also showed an increase in temperature during the second trimester of pregnancy. The calculated mean temperatures were 33.92 °C and 34.01 °C for the left and right sides, while they were lower in trimester 1 (0.66 °C for the left side and 0.89 °C for the right side, respectively). For the lower back area, greater differences in mean ∆T_L/R_ temperatures were observed for the left and right sides than for the upper back in all three trimesters. The asymmetries observed in this area were more pronounced, with a maximum value of ∆T_L/R_ = 0.35 °C recorded in the third trimester of pregnancy. It should be noted that these results represent average values, with some female volunteers showing significantly higher thermal asymmetries, as shown in Table 2.

**Figure 6:**
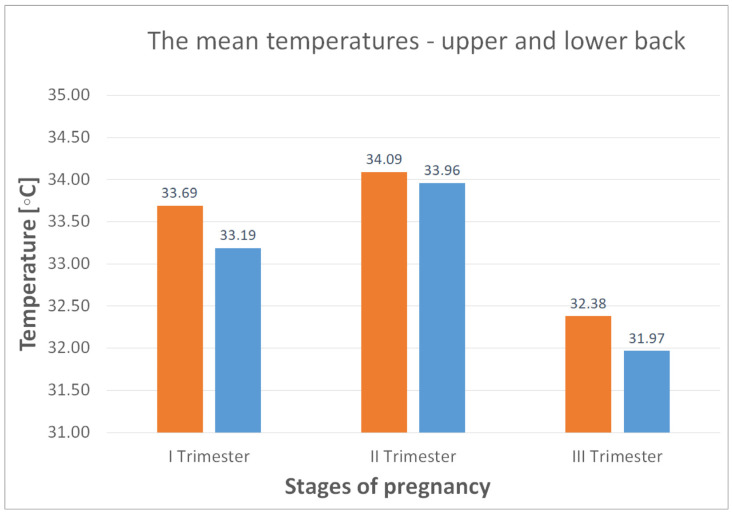
The mean temperatures recorded for the dorsal upper (orange) and lower (blue) spinal regions during the first, second and third trimesters.

**Figure 7:**
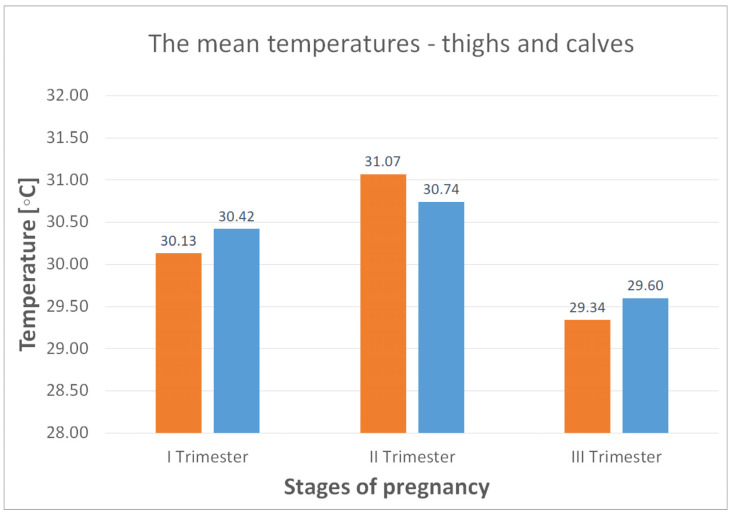
The mean temperatures recorded for the thighs (orange) and calves (blue) during the first, second and third trimesters.

As illustrated in Table 3 and Figure 6 and Figure 7, temperature variations were measured in the lumbar region and the thigh and calf areas over the three trimesters of pregnancy. The analysis reveals that the highest temperatures are observed in the second trimester.

To better illustrate the changes in average body temperatures during pregnancy, Figure 6 and Figure 7 show the temperature changes in the different parts of the body, i.e., the lower and upper back, thighs and calves, during the three trimesters of pregnancy.

The average body temperature in the upper and lower back is 33.69 and 33.19 °C, respectively (first trimester of pregnancy, Figure 6). Noticeable changes in body Tsk were noted between the first and second trimester of pregnancy, when the mean Tsk increased by 0.4 °C in the upper back and 0.77 °C in the lower back. However, between the second and third trimester, the changes in mean Tsk were significantly greater, with a decrease of 1.85 °C for the upper back and 1.95 °C for the lower back. A similar trend of an increase in mean Tsk between the first and second trimesters and a decrease between the second and third trimesters is seen for the thighs and calves, with values of 0.94 °C (increase) and 1.73 °C (decrease) for the thighs and 0.32 °C (increase) and 1.14 °C (decrease) for the calves, respectively. In summary, the second trimester showed a rise in mean Tsk in all body regions by an average of 0.61 °C. In contrast, the third trimester showed a significant decrease in mean Tsk values, with an average of 1.67 °C.

However, after analyzing the mean Tsk values for the right and left sides of the body of the female volunteers tested, the most remarkable asymmetry in the distribution of this temperature was obtained for the calves (∆T_mean_ = 0.49 °C) in the third trimester of pregnancy (Table 3). The difference between the ∆T_mean_ values for the thighs is also considerable at 0.25 °C. The asymmetries in the upper and lower back are small and within the range of 0.02 to 0.13 °C.

Despite the limited number of studies conducted up to this point, it was considered appropriate to perform a preliminary statistical analysis to assess the trends observed in the thermal images obtained for the initial group of patients studied.

A repeated-measure analysis of variance (ANOVA) was conducted, revealing a significant impact of gestational stage on the temperature variability of selected posterior body regions. Statistically significant differences in the average temperatures of these regions between successive trimesters were observed on both the right and left sides of the body (right side: F = 25.08, *p* < 0.001; left side: F = 29.15, *p* < 0.001, with the sphericity assumed and then with the application of the Greenhouse-Geisser, Huynh-Feldt, and Lower-bound corrections). The analysis also revealed significant differences in average temperature among the examined posterior regions.

The effect was particularly marked on the right (F = 44.72; *p* < 0.001) and the left side of the body (F = 40.29; *p* < 0.001). This temperature disparity was particularly evident in thermal images, when comparing the temperature of the back to that of the lower limbs (Figure 2, Figure 3, Figure 4 and Figure 5). Interaction analysis demonstrated that temperature fluctuations over time are independent of the specific body region under investigation (right side: F = 0.823; *p* = 0.61; left side: F = 0.52; *p* = 0.869). Using trend models was instrumental in determining the nature of temperature changes over time. The statistical significance of both the linear and quadratic components indicates a regular, non-random pattern of temperature fluctuations between gestation trimesters (right side: linear trend: F = 27.13; *p* < 0.001; quadratic trend: F = 22.65; *p* < 0.001; left side: linear trend: F = 26.47; *p* < 0.001; quadratic trend: F = 32.02; *p* < 0.001). Subsequent post hoc tests were conducted to ascertain which trimesters exhibited substantial fluctuations in the surface temperature of the chosen body’s posterior regions. The analysis revealed significant variations in temperature between trimesters 1 and 3 and 2 and 3 (*p* < 0.001), while no such differences were observed between trimesters 1 and 2 (*p* > 0.15). A detailed comparison of body regions was undertaken, which revealed significant temperature differences with a post hoc test. The thighs and calves exhibited significantly lower temperatures compared to the trunk areas. For instance, comparing the temperatures between the thigh and upper back on the right side revealed an average temperature difference of 3.1 °C (*p* < 0.001). Similarly, comparing the calves and lower back temperatures showed an average temperature difference of 2.1 °C (*p* < 0.001). Post hoc tests also demonstrated a significant temperature difference between the lower and upper back on the right side of the body. The mean temperature difference between these regions was 1.04 °C, which was statistically significant (*p* = 0.022). A similar comparison of temperatures for the left side did not show statistical significance.

A multivariate analysis of variance (MANOVA) was also performed to assess the significance of temperature changes in selected posterior body regions in women throughout the entire pregnancy duration. The indicators employed in this study, including Hotelling’s Trace, Roy’s Root, and Wilks’ Lambda, along with the most conservative indicator, Pillai’s Trace, have provided unequivocal confirmation of the significance of global changes in the distribution of body temperature in women throughout the entire period of pregnancy, where Wilks’ Lambda = 0.415 (right); 0.376 (left); *p* < 0.001; Pillai’s Trace = 0.585 (right); 0.624 (left); *p* < 0.001.

## 4. Discussion

During pregnancy, numerous changes occur not only in the reproductive organs but also in other organs and systems, including the endocrine, circulatory, coagulation, and fibrinolysis systems, respiratory, urinary, thyroid organs, and posture [27,28,29]. The hormones that are active from the outset of pregnancy, particularly relaxin and estrogen, induce a state of relaxation in the ligaments, joint capsules, and tendons, consequently engendering a decline in the stability of the entire body whilst concomitantly augmenting its flexibility. Concurrently, the body’s center of gravity shifts, engendering alterations in the pregnant woman’s posture and movement. It is well established that the paraspinal muscles undergo increased contraction, leading to discomfort. This phenomenon is further exacerbated by the forward pull of the abdomen, which contributes to the onset of neck, lumbar, or sacral pain [30,31,32].

The present study’s findings agree with these facts and reveal an asymmetry in the distribution of the mean temperature on the skin surface, which is most likely due to these changes. A general increase in mean Tsk values was observed in the second trimester, and a decrease in the third trimester, for all regions measured. It is important to note that a shift in the center of gravity and posture can result in heterogeneous loading of the lower limbs. In the present study, the observed physiological changes are primarily reflected in differences in mean Tsk measured in the calf area. This phenomenon can be attributed to the presence of a temperature difference of nearly 0.5 °C between the right and left sides of the body in this area during the third trimester of pregnancy. It is well established that the volume of the uterine muscle increases in the second trimester of pregnancy, resulting in increased compression of the inferior vena cava. This, in turn, leads to a decrease in venous return, increasing venous pressure within the lower limbs. It is recognized that compression of the inferior vena cava and its branches can reduce blood flow to the kidneys, the fetus-placental unit and lower body tissues. The decrease in systemic vascular and pulmonary resistance is most pronounced around 16 weeks of gestation when the smallest values are recorded. The dilatation of the venous compartment, including the end capillaries, then exceeds 150% of the baseline value. These changes increase the risk of slower metabolite absorption and, in more severe cases, venous insufficiency under conditions of vascular occlusion [27,33,34]. Consequently, monitoring the temperature of the lower limb areas becomes essential for obtaining additional information on their condition. Also, the changes in Tsk distribution observed in the thermograms can be connected with an increase in body mass and water retention. Thermal symmetry plays a significant role in muscle tension changes, so it plays a role in the fetus’s position and growth.

The analysis of the results reveals that the upper and lower back exhibit the highest average temperature during the second trimester of pregnancy (34.09 °C and 33.96 °C, respectively). This is most likely attributable to the substantial growth of the breasts, the rapid development of the fetus, and the consequent expansion of the woman’s abdomen. The compensatory response may involve the generation of force by the muscles of the back and spine to support these additional loads. Changes in the body’s center of gravity and rapid physiological changes occurring during this period may explain the occurrence of increased body temperature in the second trimester. The second trimester is often characterized by pain in patients’ L3–L5 areas of the spine, which is caused by the body’s adaptation to the changes occurring in the muscles, among other things. Moreover, the fetal position should be considered due to its influence on muscle tension and the spine. Numerous scientific studies confirm that a significant proportion of pregnant women experience lower back pain at some point during pregnancy [5,27,35,36]. The etiology and pathogenesis of these ailments remain to be fully elucidated. Most hypotheses are predicated on the premise that they result from increased body weight resulting from individually variable amounts of stored body fat, baby size, placenta, uterus and fluid retention, and decreased stability of the iliac rim associated with hormonal changes during pregnancy [27,37,38]. Non-genetic factors, such as diet and physical activity, also influence the degree of weight gain. Several studies have shown that regular exercise during pregnancy significantly reduces the intensity of back, lower limb, or pelvic pain in regularly exercising pregnant women, compared to non-exercising women [27,39,40,41].

Preliminary statistical analyses indicate that the temperature of the posterior body surface of women undergoes significant changes during pregnancy, particularly between the third trimester and the others. Subsequent post hoc tests indicated that the lower limbs exhibited significantly lower temperatures than the upper and lower back. This finding aligns with the expected physiological thermal distribution across the body surface. The study results indicate that alterations in body surface temperature are predominantly global in nature and consistent across almost all the body regions analyzed during pregnancy.

Interestingly, despite the generally symmetrical temperature distribution pattern on both sides of the body, post hoc tests indicated a unilateral temperature difference between the upper and lower back on the right side (*p* = 0.02), which was not observed on the left side. This result may indicate the presence of local thermal asymmetry within the paraspinal region of the back. Such a thermal effect may indicate the presence of unilateral physiological adaptations or functional loads, potentially related to differences in muscle activity, blood supply, or unilateral environmental influences. This phenomenon’s unilateral nature enhances the significance of our observations. It highlights the importance of incorporating a more extensive set of clinical parameters into the medical history. These additional parameters should include information on sports activities, previous injuries, rehabilitation treatments, pain complaints, and current spinal curvatures.

Asymmetrical body temperature distribution is sometimes interpreted in the literature as the result of unilateral muscle tension, postural asymmetry, different loads on body segments, or differences in blood supply. Minor temperature differences between sides may have functional significance, especially in individuals subjected to unilateral loads or in cases of muscle imbalance [10,22,24,25,30,31,34,42]. Despite the generally symmetrical temperature distribution in other body regions examined, this single finding may indicate subtle adaptive or compensatory changes in potential clinical significance.

This study has several significant limitations that should be acknowledged. First, the sample size was relatively small (n = 34), and some participants were examined in more than one trimester, which may have influenced the objectivity and generalizability of the findings. Second, we did not control for several potential confounding variables, such as maternal age, parity, body mass index (BMI), or comorbidities, which could affect the results. Finally, due to the preliminary nature of the study and the limited cohort size, we could not perform detailed statistical analyses, including correlations between temperature changes and self-reported symptoms.

Another limitation of this preliminary study is that we could not entirely exclude the influence of potential infections or hormonal disorders (e.g., thyroid diseases) on the results. Participants with diagnosed acute infections were excluded based on medical interviews, but no laboratory testing of inflammatory markers or hormonal status was performed.

Future studies, including larger and more diverse populations (also a control group), are planned and will allow us to address these limitations by incorporating these confounding variables into the analysis and performing more robust statistical evaluations.

Simultaneously, it is imperative to emphasize that a particular advantage of our study is the analysis of a larger body surface in all three stages of pregnancy, in contrast to previous work on this topic, which mainly focused on selected body parts. This is the first comprehensive approach, potentially facilitating early detection of physiological deviations and targeted monitoring of pregnant patients.

## 5. Conclusions

Thermal imaging examinations conducted during pregnancy offer valuable insights into the physiological changes in a pregnant woman’s body. Our studies demonstrated noticeable thermal alterations in the bodies of pregnant women, particularly in the spinal regions and lower limbs. The highest mean temperature values were documented in the second trimester of pregnancy, which may be associated with intensive breast growth, fetal development and the body’s adaptation to metabolic and circulatory changes.

The analysis of the thermograms revealed asymmetries in the temperature distribution mostly within the lower limb region, which may indicate modifications in musculoskeletal and circulatory load. The most considerable fluctuations in body surface temperature were observed between the second and third trimesters of pregnancy, in areas exhibiting a high physiological load, including the upper back (1.85 °C), lower back (1.95 °C), thighs (1.73 °C) and calves (1.14 °C).

Thermal imaging provides a safe, painless and effective method of monitoring physiological changes in a pregnant woman’s body. Its use, in combination with other diagnostic techniques, allows early identification of potential health complications, improving the quality of prenatal care. Typical symptoms of this condition include swelling and discomfort, particularly in the lower limbs. Employing thermal imaging technology facilitates the evaluation of such symptoms, thereby indicating areas requiring special attention. The present study revealed a considerable asymmetry in calf and thigh strain, with a temperature difference of 0.49 °C and 0.25 °C, respectively, in the third trimester of pregnancy. Furthermore, thermal imaging facilitates blood circulation assessment, helping monitor conditions such as varicose veins and thrombosis, which pose a risk to both mother and fetus.

## Figures and Tables

**Table 1:** Measured mean surface temperatures of the upper back and the difference in temperature between the body sides of the subjects in the first, second and third trimesters of pregnancy.

	I Trimester			II Trimester			III Trimester		
No.	Left Paraspinal Part—Mean Temperatures [°C]	Right Paraspinal Part—Mean Temperatures [°C]	Temperature Difference ∆T_L/R_ [°C]	Left Paraspinal Part—Mean Temperatures [°C]	Right Paraspinal Part—Mean Temperatures [°C]	Temperature Difference ∆T_L/R_ [°C]	Left Paraspinal Part—Mean Temperatures [°C]	Right Paraspinal Part—Mean Temperatures [°C]	Temperature Difference ∆T_L/R_ [°C]
1	33.2	33.6	0.4	34.8	34.7	0.1	33.1	33.1	0
2	33.2	33.2	0	34	34.1	0.1	32.1	31.9	0.2
3	34	33.9	0.1	35.7	35.5	0.2	31	30.7	0.3
4	32.1	32.5	0.4	35.1	35.3	0.2	33.9	33.8	0.1
5	34.3	34.3	0	34.6	34.8	0.2	32.3	32.4	0.1
6	34.6	34.5	0.1	32	32	0	33.1	32.8	0.3
7	33.6	33.4	0.2	31.7	31.7	0	30.7	30.5	0.2
8	33	32.9	0.1	34.6	34.7	0.1	30	29.7	0.3
9	35	35.2	0.2	34.7	34.5	0.2	34.6	34.4	0.2
10				35.4	35.4	0	32.3	32.4	0.1
11				31.9	31.7	0.2	32.1	32.3	0.2
12				34.6	34.6	0			
13				35.2	35.4	0.2			
14				32.9	33	0.1			
Average value for all female volunteers	33.67	33.72	0.17	34.09	34.1	0.11	32.29	32.18	0.18

**Table 2:** Measured mean values of lower back surface temperatures and the temperature difference between the body sides of the subjects in the first, second and third trimesters of pregnancy.

	I Trimester			II Trimester			III Trimester		
No.	Left Paraspinal Part—Mean Temperatures [°C]	Right Paraspinal Part—Mean Temperatures [°C]	Temperature Difference [°C]	Left Paraspinal Part—Mean Temperatures [°C]	Right Paraspinal Part—Mean Temperatures [°C]	Temperature Difference [°C]	Left Paraspinal Part—Mean Temperatures [°C]	Right Paraspinal Part—Mean Temperatures [°C]	Temperature Difference [°C]
1	32.50	32.10	0.40	34.70	34.80	0.10	31.80	32.00	0.20
2	32.90	33.00	0.10	33.60	33.80	0.20	31.90	31.50	0.40
3	32.80	33.00	0.20	35.60	35.80	0.20	32.80	32.10	0.70
4	31.60	32.30	0.70	35.10	35.20	0.10	33.20	32.80	0.40
5	34.00	33.80	0.20	34.20	34.50	0.30	31.10	31.10	0.00
6	34.70	34.60	0.10	32.00	32.50	0.50	32.00	31.80	0.20
7	34.00	33.10	0.90	32.40	32.30	0.10	30.90	31.10	0.20
8	31.80	31.30	0.50	34.20	34.20	0.00	30.30	30.90	0.60
9	35.00	34.90	0.10	34.20	34.20	0.00	33.30	33.20	0.10
10	33.26	33.12	0.13	35.00	35.40	0.40	31.70	32.50	0.80
11				32.20	32.30	0.10	33.20	33.00	0.20
12				34.00	33.80	0.20			
13				35.20	35.30	0.10			
14				32.50	32.00	0.50			
Average value for all female volunteers	33.26	33.12	0.33	33.92	34.01	0.20	32.02	32.00	0.35

**Table 3:** Measured mean temperature values of upper back, lower back, thighs and calves in all trimesters of pregnancy for all measured areas.

	I Trimester		II Trimester		III Trimester	
Body Areas Examined	The Average Value for Both Sides of the Body Tested [°C]	Difference Between Average Temperatures ∆T_mean_ [°C]	The Average Value for Both Sides of the Body Tested [°C]	Difference Between Average Temperatures ∆T_mean_ [°C]	The Average Value for Both Sides of the Body Tested [°C]	Difference Between Average Temperatures ∆T_mean_ [°C]
Thighs	30.13	0.18	31.07	0.02	29.34	0.25
Calves	30.42	0.27	30.74	0.12	29.60	0.49
Upper paravertebral part	33.69	0.06	34.09	0.01	32.24	0.11
Lower paravertebral part	33.19	0.13	33.96	0.09	32.01	0.02

## Data Availability

The data supporting the conclusions of this article could be made available by the authors upon reasonable request.
